# Unscented Kalman filter with parameter identifiability analysis for the estimation of multiple parameters in kinetic models

**DOI:** 10.1186/1687-4153-2011-7

**Published:** 2011-10-11

**Authors:** Syed Murtuza Baker, C Hart Poskar, Björn H Junker

**Affiliations:** 1 Systems Biology Group, Leibniz Institute of Plant Genetics and Crop Plant Research (IPK), Gatersleben, Germany

## Abstract

In systems biology, experimentally measured parameters are not always available, necessitating the use of computationally based parameter estimation. In order to rely on estimated parameters, it is critical to first determine which parameters can be estimated for a given model and measurement set. This is done with parameter identifiability analysis. A kinetic model of the sucrose accumulation in the sugar cane culm tissue developed by Rohwer et al. was taken as a test case model. What differentiates this approach is the integration of an orthogonal-based local identifiability method into the unscented Kalman filter (UKF), rather than using the more common observability-based method which has inherent limitations. It also introduces a variable step size based on the system uncertainty of the UKF during the sensitivity calculation. This method identified 10 out of 12 parameters as identifiable. These ten parameters were estimated using the UKF, which was run 97 times. Throughout the repetitions the UKF proved to be more consistent than the estimation algorithms used for comparison.

## 1. Introduction

The focus of systems biology is to study the dynamic, complex and interconnected functionality of living organisms [1]. To have a systems-level understanding of these organisms, it is necessary to integrate experimental and computational techniques to form a dynamic model [1,2]. One such approach to dynamic models is the modeling of metabolic fluxes by their underlying enzymatic reaction rates. These enzymatic reaction rates, or enzyme kinetics, are described by a kinetic rate law. Different rate laws may be used, matching the specific behaviour of the chemical reaction that is catalysed by the enzyme to the most appropriate rate law. These kinetic rate laws are formulated with mathematical functions of metabolite concentration(s) and one or more kinetic parameters. In combination with the stoichiometry of the metabolism, these kinetic rate laws define the function of the cell. In order to properly describe the dynamics, it is required to have both an accurate and a complete set of parameter values that implement these kinetic rate laws. Owing to various limitations in wet lab experiments, it is not always possible to have a measured value for all the required parameters. In these cases, it is necessary to apply computational approaches for the estimation of these unknown parameters.

In the past few years, increasing research has been made on the application of several optimization techniques towards parameter estimation in systems biology. These include nonlinear least square (NLSQ) fitting [3], simulated annealing [4] and evolutionary computation [5]. More recently, kinetic modelling has been formulated as a nonlinear dynamic system in state-space form, where the parameter estimation is addressed in the framework of control theory. One of the most widely used methods in control theory for parameter estimation is the Kalman filter [2]. However, the Kalman filter is designed for inference in a linear dynamic system, and subsequently gives inaccurate results when applied to nonlinear systems. Instead, a number of extensions to the Kalman filter have been proposed to deal with nonlinear systems. Amongst those extensions, the most widely used are the extended Kalman filter (EKF) [1] and the unscented Kalman filter (UKF) [6,7]. At the core of the UKF is the unscented transformation (UT) which operates directly through a nonlinear transformation, instead of relying on analytical linearization of the system (as performed by EKF) [7]. This nonlinear transformation gives the UKF a distinct computational advantage over the EKF. Unlike the linearization performed by the EKF, the UT does not require the calculation of partial derivatives. Furthermore, the UKF has the accuracy of a second-order Taylor approximation, while the EKF has just a first-order accuracy [7]. Overall, the UKF has been found to be more robust and converges faster than the EKF due to increased time update accuracy and improved covariance accuracy [8].

Nevertheless, parameter estimation is highly dependent on the availability and quality of the measurement data. Owing to the lack of measurement data collected from wet lab experiments, it is difficult to obtain reliable estimates of unknown kinetic parameter values. As a result, it is crucial to be able to determine the estimability of the model parameters from the available experimental data. Parameter identifiability tests are carried out to find out the estimable parameters of the model using the available experimental data and to rank these parameters based on how sensitive the model is with respect to a change in these parameters. The rank is directly proportional to the impact that the corrseponding parameter has on the system output and its ability to capture the important characteristics of the system [9]. In this article, we investigated parameter identifiability using a sensitivity-based orthogonal identifiability algorithm proposed by Yao et al. [10] with the UKF as the method for parameter estimation in a nonlinear biological model.

In the Kalman filter method, identifiability is addressed with the view of observability [2]. A system is said to be observable if the initial state can be uniquely identified from the output data at any given point in time [11]. However, most observability analysis methods work by first calculating an analytical solution of the system, which is not possible if the system is considerably large and nonlinear. The novelty of this study lies in the fact that we propose to embed a sensitivity-based method for identifiability analysis into the UKF during the estimation of the parameter. The central difference (CD) method was used to calculate the sensitivity coefficient, where the step size is taken as the square root of the variance generated by the UKF at each step of its iteration. For the implementation, testing and validation of these methods, we have taken the sucrose accumulation in the sugar cane culm model published by Rohwer et al. [12].

## 2. Methods

### 2.1. Problem statement

In this article, the biological model is described as a state-space model which is a convenient way to describe a nonlinear system in terms of first-order differential equations. The model can be represented as

(1) $$Ẋ=f\left(X,\theta ,t\right),\;X\left(t_{0}\right)=X_{0}$$

where *f *is the nonlinear function describing the reactions, each of which is made up of the sum or difference of individual rate laws (see Additional file 1, Supplementary data). The vector *X *is the state vector of the model, values of which are the metabolite concentrations, and *X*_0 _is the initial state vector at time *t*_0_. The vector *θ *contains the unknown rate coefficients, such as Michaelis-Menten parameters, which we want to estimate. As the parameters are constant, it is possible to construct an augmented state vector by treating *θ *as additional state variables with zero rate of change, $\dot{\theta }=0$. The output vector *Y *is the output signal vector, or the vector of the quantities that can be measured from biological experiments,

(2) Y=g(X)

This output signal is related to the state through a function *g *that encodes the relationship between the state of the system, *X*, and the measurement data at any given time. Having the measurement data, we try to estimate the parameter values by minimizing the distance between the measured data (actual) and the model data (estimated).

Parameter identifiability attempts to answer the question of whether or not the parameters of a given model can be uniquely identified with the given level of experimental data. Only if identifiability can be assured for the combined set of model parameters and measurement data, is it then reasonable to continue the estimation process. In this article, we simulate the measurement data from the model. This synthetic data is derived by combining the simulated data with random noise to develop a realistic experimental dataset [13].

Several theories of identifiability analysis exist, the most widely applied of which are introduced, and one of those is chosen for evaluation. A model is *globally identifiable *if a unique value can be found for each of the model parameters that reproduce the experimental data. On the other hand, if a finite number of sets of parameter values can be found, which reproduce the experimental data, then the model is called *locally identifiable*. Finally, the model is said to be *unidentifiable *if there exist an infinite number of possible parameter sets that can reproduce the experiment.

Two classes of identifiability analysis arise depending on the availability of prior information on the parameter data. The first is structural identifiability analysis and the second is posterior identifiability analysis [14]. For structural identifiability analysis, no prior information about the parameter values are required, whereas for posterior identifiability analysis prior information about the parameter values are needed. On the other hand, structural identifiability analysis is highly restricted to either linear models or for the nonlinear case, small models with less than ten states and parameters [15]. For our analysis, we used a posterior identifiability approach, specifically local at-a-point identifiability (a specific method of locally identifiable modelling [14]).

For large nonlinear models, posterior identifiability methods are feasible. Yao et al. [10] developed an orthogonal-based parameter identifiability method using a scaled sensitivity matrix. Jacquez et al. [16] developed a method based on correlation, and Degenring et al. [17] developed a method based on principal component analysis. All of these methods are local at-a-point identifiability analysis methods and perform similarly with nonlinear biological models [14]. For our approach, we have chosen the orthogonal-based method because of its ease of implementation and straightforward analysis. We applied this orthogonal method of parameter identifiability to determine the set of identifiable parameters and then applied the UKF to perform the estimation of these unknown parameters.

### 2.2. Unscented Kalman filter

The UKF is based on a statistical linearization technique. Starting with a nonlinear function of random variables, a linear regression between *n *points is drawn from the prior distribution of the random variables. This technique gives a more accurate result than analytical linearization techniques, such as Taylor series linearization, as it considers the spread of the random variables [18].

A Kalman filter is composed of a number of equations which estimate the state of a process by minimizing the covariance of the estimation error. Kalman filters work in a predictor-corrector style, whereby they first predict the process state and covariance at some time using information from the model (prediction) and then improve this estimate by incorporating the measurement data (corrector). UKF is itself an extension of the UT [7], a deterministic sampling technique which implements a native nonlinear transformation to derive the mean and covariance of the estimates. This transformed mean and covariance are then supplied to the Kalman filter equations to estimate the state variables.

In order to implement the UKF for parameter estimation, we use the discrete time description of the continuous time process. The system at discrete time points *t*_1_,...,*t_k _*is described as

(3) $$\begin{array}{c} X\left(t_{k+1}\right)=f\left(X\left(t_{k}\right)\right)+w\\ Y\left(t_{k}\right)=h\left(X\left(t_{k}\right)\right)+v \end{array}$$

where *f*, *X *and *Y *are as described in (1) and (2), *h *describes an incomplete and noisy observation model, and both *w *and *v *are uncorrelated white noises of the system and measurement model, respectively. During the UT, sigma points, a minimal set of sample points about the mean, are calculated to capture the statistics of the state model. The sigma points are calculated according to the following equation:

(4) $$X_{i}=\left\{\begin{array}{ccc} \bar{x} & \bar{x}+\gamma \sqrt{P_{x}} & \bar{x}-\gamma \sqrt{P_{x}} \end{array}\right\}$$

where $\gamma =\sqrt{L+\lambda }$, *L *is the dimension of the augmented state; *λ *is the composite scaling parameter; and *P_x _*is the system uncertainty. The sigma points are then transformed through the nonlinear function *f*, *Y_i _*= *f*(*X_i_*). The mean and covariance are then calculated according to Equation 5:

(5) $$\begin{array}{c} ȳ=\sum \overset{m}{\underset{i}{W}}Y_{i}\\ P_{y}=\sum \overset{c}{\underset{i}{W}}\left(Y_{i}-ȳ\right){\left(Y_{i}-ȳ\right)}^{T} \end{array}$$

where $W_{i}^{m}$ and $W_{i}^{C}$ are the corresponding weights to calculate, respectively, the mean and covariance of the state. The transformed mean and covariance are then fed into the standard Kalman filter equations to make the process estimation.

### 2.3. Orthogonal-based method for parameter identifiability

The orthogonal method for parameter identifiability proposed by Yao et al. [10] is a method based on sensitivity analysis. Sensitivity analysis is used for determining the relationship between a change in the parameters and the corresponding change to the system. Sensitivity coefficients, the elements of the sensitivity matrix, are calculated through the partial derivative of the model states with respect to the model parameters. In the orthogonal method, this sensitivity coefficient is calculated local at-a-point. Identifiability analysis describes two things, first which of the parameters have high sensitivity to the system output and then which of the parameters are linearly independent. The method iterates over the columns of the sensitivity matrix *Z *to select the column with the highest sum of squared value. Since each column corresponds to a single parameter, this corresponds to the parameter that has the highest impact on the model output. This column is added to the matrix *X_L _*(*L *being the iteration number), in the order of the highest to the lowest sensitivity. To make the adjustment of the net influence of each of the remaining parameters on the already selected parameters, all of the original columns of *Z *are being regressed on the column associated with the most estimable parameter (denoted ${Ẑ}_{L}$). A residual matrix *R_L _*is calculated to measure the orthogonal distance between *Z *and the regression matrix ${Ẑ}_{L}$. The column having the highest sum of squared value in the residual matrix *R_L _*is chosen to be the next most estimable parameter. The steps are repeated until a specific cutoff value of *R_L _*is reached or until all the parameters have been selected as identifiable. The algorithm is as follows:

1. Calculate the sensitivity coefficient matrix *Z*.

2. Calculate the sum of squared values of the *Z *matrix and choose the highest column to be the most estimable one.

3. Mark the column as *X_L _*where $L\in \left\{\text{1,}\ldots ,n_{p}\right\}$.

4. Calculate an orthogonal projection ${Ẑ}_{L}$ for the column that exhibits the highest independence to the vector space *V *spanned by *X_L_*.

$${Ẑ}_{L}=X_{L}{\left(X_{L}^{T}X_{L}\right)}^{-1}X_{L}^{T}Z$$

5. The residual matrix, $R_{L}=Z-{Ẑ}_{L}$, is calculated as a measure of independence.

6. The sum of squares values is calculated for each column of the *R_L _*matrix, resulting in the vector *C_L_*, and the column corresponding to the largest sum of squares is chosen for the next estimable parameter.

7. Select the corresponding column in *Z *and augment the matrix *X_L _*by marking the new column.

8. Iterate steps 4-7 until the cutoff value is reached or until all of the parameters are selected to be identifiable.

The sensitivity matrix *Z *is defined as

(6) $$Z=\frac{\partial X}{\partial \theta }=\left[\begin{array}{cccc} z_{11} & z_{12} & \cdots \; & z_{1n}\\ z_{21} & z_{22} & \cdots \; & z_{2n}\\ \vdots & \vdots & \ddots & \vdots \\ z_{n1} & z_{n2} & \cdots \; & z_{nn} \end{array}\right]$$

An analytical solution of the state-space equation is very rare for nonlinear biological systems. As a result, the matrix *Z *must be solved numerically for each iteration. To do this, the CD method was applied. This method uses the finite difference approximation, where the sensitivity coefficient *z_i,j _*is calculated from the difference of the perturbed solutions around the nominal value.

(7) $$z_{i,j}\left(t\right)=\frac{x_{i}\left(\theta_{j}+\Delta \theta_{j},t\right)-x_{i}\left(\theta_{j}-\Delta \theta_{j},t\right)}{2\Delta \theta_{j}}$$

In this approach, the choice of step size, Δ*θ_j_*, is critical as numerical values obtained with this method depend highly on the value of the step size. The square root of the variance generated by UKF at each step of its iteration was used as the step size, which gives $\Delta \theta_{j}=\sqrt{Px_{j,j}}$[19]. This choice is made to ensure that the step size remains variable with each recursive step, as well as within the feasible parameter range of the perturbed system. It has been shown that the approximation error gets smaller linearly as step size becomes smaller [20]. Parameters are maintained within one standard deviation (the approximation error), and thus, they have a higher probability in comparison to parameters outside of this range. Furthermore, with each recursion the availability of new information during the parameter estimation in UKF correlates to a general decrease in the uncertainty within the system [21], making the standard deviation a feasible choice for the step size.

## 3. Analysis

### 3.1. Model setup

**Figure 1:**
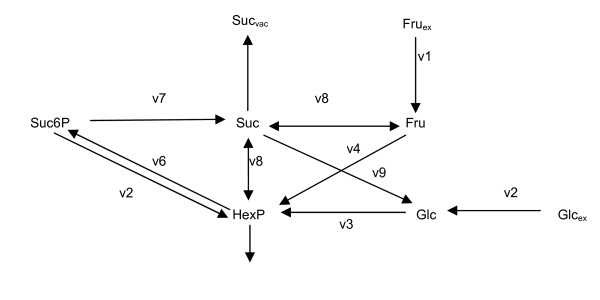
**Schematic diagram of the case study model--the sucrose accumulation in sugar cane culm tissue**.

The sucrose accumulation in sugar cane culm tissue was chosen as the study model for both the identifiability analysis and the parameter estimation. The model, the identifiability analysis and the parameter estimation were all implemented using MATLAB (R2009b) numerical toolkit.^a ^All the parameter values are known *a priori *[12]. The schematic diagram of the model is given in Figure 1.

A set of ODEs are generated from the sugarcane model to formulate a mathematical model of the network. The system has five metabolites that are free to change and three that remain fixed, with a total of 54 parameters. All the 54 known parameters were used initially for developing the synthetic measurement data. In testing both the identifiability analysis and the parameter estimation, 12 of these parameters have been assumed to be unknown (see Table 1) and initialized to random numbers between zero and one.

**Table 1:** Parameters chosen to be unknown, and their corresponding rank, or position in the residual matrix

Parameter number	Parameter name	Identifiability rank
1	*v1.Ki1Fru*	8
2	*v2.Ki2Glc*	9
3	*v3.Ki3G6P*	6
4	*v3.Ki4F6P*	Not Identifiable
5	*v6.Ki6Suc6P*	3
6	*v6.Ki6UDPGlc*	1
7	*v6.Vmax6r*	2
8	*v6.Km6UDP*	7
9	*v6.Km6Suc6P*	4
10	*v6.Ki6F6P*	5
11	*v11.Vmax11*	10
12	*v11.Km11Suc*	Not identifiable

Parameters 4 and 12 have no rank, as they were found to be unidentifiable

### 3.2. Results

We start with the ODEs by first integrating them over the time interval [0 *T*] where *T *= 5000 with all the known parameters to generate the synthetic measurement time series data. We choose the final time point to be the time when the system reaches its steady state. The MATLAB function ode45 (a numerical Runge-Kutta method for numerical integration) was used for solving the ODE. The synthetic measurement data were created through the inclusion of a small random uncorrelated white noise to the observation. During the simulation, the measurement data are sampled at a fixed interval of Δ*t *= 0.2, to collect fixed time points.

In order to make a fair comparison of the UKF to other methods of parameter estimation, the identifiability analysis was performed separately. This should not affect the advantage of integration of identifiability with estimation, but in fact detract from it, as it gives the other estimation algorithms an effective headstart.

Therefore, we first performed the identifiability analysis, to determine which parameters could be estimated. The 12 parameters assumed to be 'unknown' were initialized as previously described. The identifiability analysis revealed that 10 out of the 12 parameters were identifiable (see Table 1). In the method proposed by Yao et al. [10], heuristics were used for determining the condition to stop the selection of identifiable parameters. We followed the same procedure laid out in Yao et al. [10], and found the condition for a reasonable stopping criterion to be Max(*C_L_*) < 0.004.

The UKF parameter estimation algorithm was repeated for 97 runs to provide statistics of the estimation. In order to compare the parameter estimation methods as these parameters have the least effect on the system, we keep the nonidentifiable parameters fixed to their known values [12]. In general, however, these parameters would not be known *a priori*. In these cases, we would first try to resolve the parameter identifiability through restructuring the model and, only as a last resort, set them to fixed arbitrary values.

**Figure 2:**
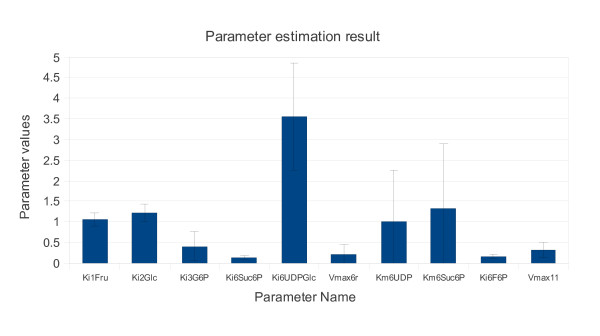
**The mean of the estimated values of the ten identifiable parameters**. The error bars indicated the standard deviation.

In all cases, the parameters are initialized to a small random number between zero and one. Throughout the simulation, the algorithm adjusts the parameter values by adjusting the covariance matrix. This is performed by comparing the measured data to the data generated from the model. The results of the parameter estimation are illustrated in Figure 2.

Though the method estimated most of the parameter values with lower standard deviation, parameters, *Km6UDP *and *Km6Suc6P*, show decidedly higher standard deviation. This high variation contradicts the evaluation of the identifiability analysis. One possible explanation is that these two parameters have some sort of a functional relationship (nonlinear) with other parameters. The orthogonal nature of the parameter identifiability approach proposed by Yao et al. can only deal with collinearity. A second possible explanation could be the local identifiability approach, as applied in this study, which by definition only ensures that the system is identifiable within a finite (but not unique) set of points in the parameter space. Individual parameters within this set could have a very large domain, resulting in a large variation within the individual parameter, i.e. the parameter is identifiable but poorly resolved.

**Figure 3:**
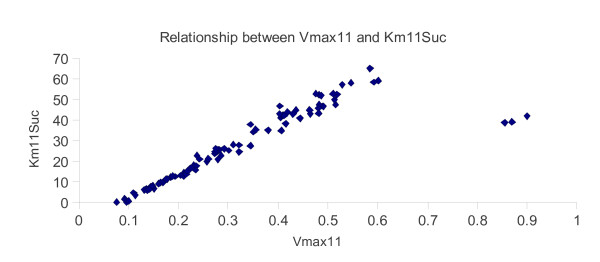
**Relationship between parameters Vmax11 and Km11Suc, via Vanted data alignment analysis**.

The two parameters 4 (*Ki4F6P*) and 12 (*Km11Suc*) were found to be nonidentifiable. This means that an infinite number of possible solution sets could be found when these parameters are included. The main reason for this is that these parameters are somehow dependent on the remaining parameters. In the case of *Km11Suc*, an exhaustive functional analysis with each of the other parameters individually found that *Km11Suc *has a strong linear relationship with parameter *Vmax11*, as illustrated in Figure 3. A similar analysis was unable to find a simple relationship between *Ki4F6P *and any one of the identifiable parameters.

To better gauge the parameter estimation of the UKF, the ten estimable parameters were similarly determined using a genetic algorithm (GA) and NLSQ. Both alternatives were implemented in MATLAB, using the default implementations and settings. A third alternative, simulated annealing, was attempted using the implementation in Copasi. However, this method on its own failed to produce usable parameters and required more than an order of magnitude longer to run. As with the UKF, 97 repetitions were performed for each of these methods.

**Figure 4:**
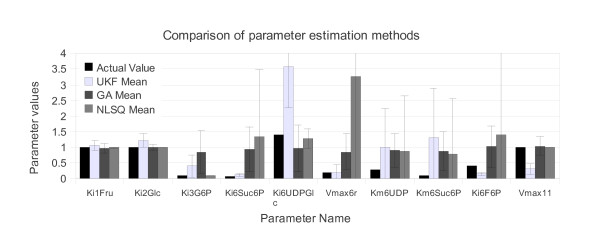
**Comparison of the actual value of the identifiable parameters to the results of the three-parameter-estimation methods**. The error bars represents the standard deviation.

The comparison of the parameter estimation methods is presented in Table 2 and Figure 4. In each case, the mean and standard deviation are calculated for the 97 repetitions, and are used for the comparison. Four values are plotted for each parameter in the bar chart of Figure 4. The first bar represents the actual value of the parameter as determined in [12]. The remaining bars represent the estimated values of the corresponding parameter, from left to right, for the UKF, the GA and the NLSQ methods. No one method correctly identifies all the ten parameters; however, the UKF consistently performs as good as or better than either GA or NLSQ.

**Table 2:** Comparison of actual parameter values and the parameter estimation results using UKF, GA and NLSQ

Parameter name	Actual value	UKF		GA		Nonlinear LSQ	
		
		Mean	SD	Mean	SD	Mean	SD
*v1.Ki1Fru*	1.00	1.06	0.15	0.97	0.15	0.99	0.007
*v2.Ki2Glc*	1.00	1.21	0.22	1.00	0.09	0.99	0.001
*v3.Ki3G6P*	0.10	0.40	0.36	0.85	0.69	0.10	0.010
*v6.Ki6Suc6P*	0.07	0.13	0.05	0.94	0.72	1.35	2.135
*v6.Ki6UDPGlc*	1.40	3.56	1.29	0.97	0.74	1.29	0.305
*v6.Vmax6r*	0.20	0.21	0.23	0.86	0.56	3.27	4.932
*v6.Km6UDP*	0.30	1.00	1.23	0.90	0.55	0.89	1.747
*v6.Km6Suc6P*	0.10	1.32	1.56	0.88	0.62	0.78	1.775
*v6.Ki6F6P*	0.40	0.15	0.05	1.02	0.67	1.40	3.875
*v11.Vmax11*	1.00	0.31	0.18	1.04	0.29	0.99	0.001

Neither the GA nor the NLSQ performed well when the parameter value fell below 1, which accounted for six out of the ten parameters. In fact, with one exception (NLSQ parameter Ki3G6P), only the UKF was able to consistently estimate smaller parameters. In fact the GA seemed to have difficulties with any parameter too far from 1, with all mean parameters falling between 0.85 and 1.04 with very small standard deviations. Similar to the GA, the NLSQ estimation shows very tight results for the parameters with value 1 (standard deviations < 0.01), and with the exception of the parameter Ki3G6P, the standard deviations increase considerably as the parameter value differs from 1 (with five of the standard deviations exceeding 100% of the parameter value). The UKF is more consistent throughout, estimating both larger and smaller values with more consistent standard deviations.

## 4. Conclusion

In order to develop dynamic models for systems biology, it is necessary to have knowledge of the underlying kinetic parameters for the system being modelled. Since it is not always possible to have this knowledge directly from experimental measurements, it is necessary to develop a method to estimate these parameter values. Furthermore, it is critical that we rely on the accuracy of these estimated values. One step towards this is the parameter identifiability which can be used to help determine if there are sufficient measurement data with which to identify the parameter(s).

In this article, we have proposed a method whereby biological systems can be viewed as a state-space system, in order to apply approaches from control theory, the UKF, to parameter estimation. However, before approaching the estimation problem, an identifiability approach proposed by Yao et al. [10] was applied to identify the parameters which cannot be uniquely estimated, based on the model structure and the measurement data. One of the benefits in integrating estimation and identifiability is the reuse of the variance generated by the UKF for the step size in the calculation of the sensitivity coefficient for identifiability.

The UKF offers many desirable traits to biological modelling, chief among them being a native nonlinear transformation [22]. The UKF is thus able to overcome one of the major bottlenecks in biological modelling, a lack of experimentally measured parameters. The UKF with identifiability analysis is particularly important in the study of kinetic networks, as a large number of parameters might be unidentifiable as these networks increase in size and complexity. Another aspect of the UKF that lends itself to kinetic models is that UKF is a time-evolution algorithm. This means that the parameter estimation with UKF is refined with each additional set of measurements, making it especially successful at estimating biochemical pathways with time series data.

In our future study, we intend to refine the methods to better identify the functional relationship(s) between parameters and quantify them. By applying the identifiability analysis, we will estimate the independent parameters and determine the dependent ones from this quantification. One other thrust of research will be in generalizing the stopping criterion for identifiability analysis. For this test model, it was found that Max(*C_L_*) < 0.004 provided the desired stopping criterion, but it is unknown if this is a model- or data-specific value.

## Abbreviations

CD: central difference; EKF: extended Kalman filter; GA: genetic algorithm; NLSQ: nonlinear least squares; UKF: unscented Kalman filter; UT: unscented transformation.

## Competing interests

The authors declare that they have no competing interests.

## Endnotes

^a^Matlab source for implementation can be made available upon request.

## Supplementary Material

Additional file 1 **Supplementary Data**. Rate laws used in this model, as developed by Rohwer et al. [12]. Click here for file
