# Supplementary material for: Unscented Kalman filter with parameter identifiability analysis for the estimation of multiple parameters in kinetic models
Source: EURASIP J Bioinform Syst Biol. 2011 Oct 11;2011(1):7. doi: 10.1186/1687-4153-2011-7 (PMC3224596; doi:10.1186/1687-4153-2011-7)
Supplement: Additional file 1 — Supplementary Data. Rate laws used in this model, as developed by Rohwer et al. [12]. [file 1687-4153-2011-7-S1.DOC]

**Supplementary Data**

Rate laws used in this model, as developed by Rohwer et al. [12].

$$v_{1}=V_{\max1}\frac{[Fru_{ex}]}{K_{m1Fru_{ex}}\left(1+\frac{[Fru]}{K_{i1Fru}}\right)+[Fru_{ex}]}$$

$$v_{2}=V_{\max2}\frac{[Glc_{ex}]}{K_{m2Glc_{ex}}\left(1+\frac{[Glc]}{K_{i2Glc}}\right)+[Glc_{ex}]}$$

$$v_{3}=V_{\max3}\frac{\frac{[Glc]}{K_{m3Glc}}\frac{[ATP]}{K_{m3ATP}}}{\left(1+\frac{[ATP]}{K_{m3ATP}}\right)\left(1+\frac{[Glc]}{K_{m3Glc}}+\frac{[Fru]}{K_{m4Fru}}+\frac{[Glc6P]}{K_{i3Glc6P}}+\frac{[Fru6P]}{K_{i4Fru6P}}\right)}$$

$$v_{4}=V_{\max4}\frac{\frac{[Fru]}{K_{m4Fru}}\frac{[ATP]}{K_{m4ATP}}}{\left(1+\frac{[ATP]}{K_{m4ATP}}\right)\left(1+\frac{[Glc]}{K_{m3Glc}}+\frac{[Fru]}{K_{m4Fru}}+\frac{[Glc6P]}{K_{i3Glc6P}}+\frac{[Fru6P]}{K_{i4Fru6P}}\right)}$$

$$v_{5}=V_{\max5}\frac{\frac{[Fru]}{K_{m5Fru}}\frac{[ATP]}{K_{m5ATP}}}{\left(1+\frac{[Fru]}{K_{i5Fru}}\right)\left(1+\frac{[Fru]}{K_{m5Fru}}+\frac{[ATP]}{K_{m5ATP}}+\frac{[Fru][ATP]}{K_{m5Fru}K_{m5ATP}}+\frac{[ADP]}{K_{i5ADP}}\right)}$$

$$\begin{matrix} v_{6}=V_{f6}\frac{[Fru6P][UDPGlc]-\frac{[Suc][UDP]}{K_{eq6}}}{[Fru6P][UDPGlc]\left(1+\frac{[Suc6P]}{K_{i6Suc6P}}\right)+K_{m6Fru6P}\left(1+\frac{[P_{i}]}{K_{iP_{i}}}\right)\left([UDPGlc]+K_{i6UDPGlc}\right)+K_{m6UDPGlc}[Fru6P]+\frac{V_{f6}}{V_{r6}K_{eq6}}\left(\begin{matrix} \\ \end{matrix}\cdots\right.} \\ \left.\cdots[Suc6P]K_{m6UDP}\left(1+\frac{[UDPGlc]}{K_{i6UDPGlc}}\right)+[UDP]\left\{K_{m6Suc6P}\left(1+\frac{[Fru6P]K_{m6UDPGlc}}{K_{i6UDPGlc}K_{m6Fru6P}\left(1+\frac{[P_{i}]}{K_{iP_{i}}}\right)}\right)+[Suc6P]\left(1+\frac{[Fru6P]}{K_{i6Fru6P}}\right)\right\}\right) \end{matrix}$$

$$v_{7}=V_{\max7}\frac{[Suc6P]}{K_{m7Suc6P}+[Suc6P]}$$

$$v_{8}=-V_{f8}\frac{[Suc][UDP]-\frac{[Fru][UDPGlc]}{K_{eq8}}}{[Suc][UDP]\left(1+\frac{[Fru]}{K_{i8Fru}}\right)+K_{m8Suc}\left([UDP]+K_{i8UDP}\right)+K_{m8UDP}[Suc]+\frac{V_{f8}}{V_{r8}K_{eq8}}\left([Fru]K_{m8UDPGlc}\left(1+\frac{[UDP]}{K_{i8UDP}}\right)+[UDPGlc]K_{m8Fru}\left(1+\frac{K_{m8UDP}[Suc]}{K_{i8UDP}K_{m8Suc}}\right)+[Fru]\left(1+\frac{[Suc]}{K_{i8Suc}}\right)\right)}$$

$$v_{9}=V_{\max9}\frac{[Suc]}{\left(1+\frac{[Glc]}{K_{i9Glc}}\right)\left(K_{m9Suc}\left(1+\frac{[Fru]}{K_{i9Fru}}\right)+[Suc]\right)}$$

$$v_{10}=V_{\max10}\frac{[Fru6P]}{K_{m10Fru6P}+[Fru6P]}$$

$$v_{11}=V_{\max11}\frac{[Suc]}{K_{m11Suc}+[Suc]}$$
